# Gold Nanoparticle-Based Enzyme-Assisted Cyclic Amplification for the Highly-Sensitive Detection of miRNA-21

**DOI:** 10.3390/bios12090724

**Published:** 2022-09-04

**Authors:** Yang Qing, Yuxing Yang, Ping Ouyang, Chenxin Fang, Haobin Fang, Yazhen Liao, Haiyu Li, Zhencui Wang, Jie Du

**Affiliations:** State Key Laboratory of Marine Resource Utilization in South China Sea, College of Materials Science and Engineering, Hainan University, Haikou 570228, China

**Keywords:** exonuclease III, miRNA-21, AuNPs, fluorescence quenching, fluorescence recovery

## Abstract

Because microRNAs (miRNAs) are biological indicators for the diagnosis, treatment, and monitoring of tumors, cancers, and other diseases, it is significant to develop a rapid, sensitive, and reliable miRNA detection platform. In this study, based on miRNA-21 detection, DNA-a with a 3′ end overhang and Texas Red fluorophore-labeled 5′ end was designed, which reacts with miRNA-21 and hybridizes with exonuclease III (Exo III), where the part connected to miRNA-21 is hydrolyzed, leaving a-DNA. At the same time, miRNA-21 is released to participate in the following reaction, to achieve cyclic amplification. a-DNA reacts with DNA-b conjugated to gold nanoparticles to achieve fluorescence quenching, with the quenching value denoted as *F*; additionally, after adding DNA-d and linked streptavidin immunomagnetic beads (SIBs), fluorescence recovery was achieved using DNA-c, with the recovered fluorescence recorded as *F*_0_. By comparing the difference in the fluorescence (*F*_0_ − *F*) between the two experiments, the amount of DNA-a hydrolyzed to produce a-DNA was established to determine the target miRNA-21 content. Under optimized conditions, by comparing the changes in the fluorescence signal, the developed strategy shows good sensitivity and repeatability, with a detection limit of 18 pM, good discriminative ability and selectivity, and promise for the early diagnosis of breast and intestinal cancers.

## 1. Introduction

MicroRNAs (miRNAs) are non-coding ribonucleic acid (RNA) molecules made up of 18–23 nucleotides [1] that are involved in stem-cell differentiation and renewal [2], viral replication [3], and tumor metastasis [4]. Taking miRNA-21 as an example, miRNA-21 is located in the 17q23.2 chromosome in humans [5]. Studies have shown that miRNA-21 is a biomarker for breast cancer [6], which, as of 2020, was the most common cancer in the world, according to global cancer statistics [7]. Moreover, miRNA-21 is also a biomarker for rectal cancer [8], which as of 2018, accounted for 6.1% of all cancers [9]. Recent studies have shown that miRNA-21 regulates other miRNAs, affecting other miRNA species [10]. Therefore, accurate detection of miRNA-21 in cells is of great value for the early diagnosis of diseases and the development of anti-cancer drugs [11].

Traditionally, miRNAs are detected by colorimetry [12], microarray technology [13], northern blotting [14], reverse transcription-polymerase chain reaction (RT-PCR) [15], electrochemical methods [16], and other techniques. However, these methods require a corresponding implementation environment. Moreover, due to the small size, low concentration, and high homology of the miRNA itself [17], it is challenging to apply these methods to detect miRNA in complex biological matrices. Therefore, there is an urgent need to develop an inexpensive, simple, highly specific, and rapid detection method for the detection of miRNA. Based on these advantages, some studies have focused on fluorescence techniques [18]. 

In recent years, amplification strategies, such as loop-mediated isothermal amplification (LAMP) [19], polymerase chain reaction (PCR) [20], hybridization chain reaction (HCR) [21], and catalytic hairpin assembly (CHA) [22], have been successfully applied in the detection of miRNAs. Among them, the excellent characteristics of enzymes, such as the excellent amplification exhibited by the cyclic reaction of enzymes, have been employed in recent years in miRNA analysis strategies based on their combination with nanomaterials. For example, Li et al. used enzyme-assisted cyclic amplification and the generation of silver nanocluster signals on DNA templates to achieve an off-fluorescence method to detect microRNA-122 [23]. Moreover, in terms of materials selection, nanomaterials have been used to construct various sensors due to their attractive properties, such as good water solubility, low toxicity, high quantum yield, and excellent biocompatibility [24]. Taking gold nanomaterials as an example, metal nanoclusters can be readily assembled from several or more atoms using easy preparation methods [25]. In addition, gold nanoparticles (AuNPs) can be chemically bonded to thiols, disulfides, and amine groups, and by exploiting these properties, can be used to design different biosensors [26]. For example, Zhang et al. [27] used gold–platinum nanoflowers as colored and catalytic markers for ultrasensitive lateral flow toward microRNA-21 detection. In addition, He et al. designed a molecular motor biosensor for the ultrasensitive detection of miRNA-155 using AuNPs and a DNAzyme linkage [28].

In this study, a fluorescent biosensor was designed for the detection of miRNA-21 based on the hydrolysis of double-stranded DNA by Exo III [29], the fluorescence burst of AuNPs [30], and excellent separation using streptavidin immunomagnetic beads (SIBs) [31]. Specifically, in the first step, the target miRNA-21 was reacted with DNA-a labeled with a fluorescent moiety and hydrolyzed from the 3′ end by the action of Exo III, followed by the addition of DNA-b conjugated to AuNPs to achieve fluorescence burst. In the second step, DNA-d and DNA-c conjugated to SIBs were added under the same conditions to achieve fluorescence recovery. In the third step, the detection of miRNA-21 was achieved by comparison of data measured twice. Finally, the sensitivity and specificity of the designed biosensor were determined experimentally.

## 2. Materials and Methods

### 2.1. Materials

All chemicals and reagents were of at least analytical reagent grade and were used as received without any further purification. Trisodium citrate was purchased from Acros Organics. Hydrogen tetrachloroaurate (III) (HAuCl_4_) trihydrate and tris (2-carboxyethyl) phosphine hydrochloride (TCEP) were purchased from McLean Biochemical Co., Ltd. (Shanghai, China). pH reference solution (4 M LiCl) and ultrapure water were purchased from Merrill Chemicals Technology Co., Ltd. (Shanghai, China). Sodium chloride (NaCl) was purchased from Aladdin Co., Ltd. (Shanghai, China). Tween-20 was purchased from KGI Biotechnology Co., Ltd. (Nanjing, China). 2-Amino-2-(hydroxymethyl)-propane-1,3-diol (Tris) was purchased from Saiguo Biotech Co., Ltd. (Guangzhou, China). Exonuclease III (ExoIII) was purchased from New England Biolabs Co., Ltd. (Guangzhou, China). Streptavidin immunomagnetic beads (SIBs) (Diameter 500 nM), phosphate-buffered saline (PBS, 10×), and all DNA and miRNA were purchased from Sangon Biotechnology Co., Ltd. (Shanghai, China) Co., Ltd. Synthesized and purified, and the sequences are listed in Table S1. In the experiments, the 5′ end of DNA-a was functionalized with Texas Red, the 3′ end of the DNA-b was a thiol, –(CH_2_)_6_– was used as a spacer, and the 3′ end of the DNA-c was biotin.

### 2.2. Apparatus

Fluorescence spectroscopy was conducted using an RF-6000 fluorescence spectrometer (Shimadzu, Japan), with fluorescence generated by Texas Red, which was used to label the 5′ end of DNA-a as the signal. The AuNPs were characterized by field-emission transmission electron microscopy (Talos F200X G2) (Thermo Scientific, UK). After ligation, the nanoparticles and DNA were separated and purified using a centrifuge (D2012, SCILOGEX Corporation, Rocky Hill, CT, USA). An ultraviolet (UV) spectrophotometer (Lambda 750 s) (PE, Waltham, MA, USA) was used to measure the absorbance of the AuNPs.

### 2.3. Preparation of the Reagents

Custom DNA was diluted to 100 µmol L^−1^ using enzyme-free sterile ultrapure water, and miRNA was diluted to 20 µmol L^−1^ by adding DEPC (diethyl decarbonate) water. The buffer used in the experiments was PBS (1X), which was stored in the refrigerator at 4 °C. Store Exo III at −20 °C. When using, configure the corresponding enzyme buffer (NEbuffer) to 1X and the concentration to be 2.5 UµL^−1^. TTL buffer is composed of 1 M LiCl reference solution, 100 mM Tris, and 0.1% Tween-20, and stored at 4 °C due to the shelf life of the reagent. Other reagents were used as required according to their instructions.

### 2.4. Synthesis of the AuNPs

According to the protocols of Amini et al. [30] and Shi et al. [32], AuNPs were synthesized by reduction using citrate. Briefly, HAuCl_4_ (1 mM, 100 mL) was placed in a beaker, stirred at 700 rpm, and heated until its boiling point was reached, at which point a solution of sodium citrate (38.5 mM, 5 mL) was quickly added dropwise and the color of the reaction solution changed from yellow to purple within a few seconds. After approximately 2 min, the purple solution turned dark red, indicating the formation of AuNPs. The reaction was heated at 100 °C for a further 20 min at 700 rpm. Finally, the AuNPs were cooled to room temperature by turning off the heater while maintaining a constant stirring speed of 700 rpm. The colloidal AuNPs were then stored in a refrigerator set at 4 °C until use. The maximum absorbance of the AuNPs was measured using a UV spectrophotometer (Lambda 750s). The structure and size of the AuNPs were measured by transmission electron microscopy (TEM).

### 2.5. Functionalization of the AuNPs

The AuNPs were functionalized according to a method reported by Tang et al. [33], with slight modification. DNA-b (1 µL, 100 µM) in PBS (20 µL, 1×, pH 7.4) was mixed with TCEP (5 µL, 10 mM) and activated for 1 h. Then, an aliquot of the colloidal solution of AuNPs synthesized above (1 mL) was added to this solution, and the mixture was incubated at room temperature for 16 h. Next, a total of 200 µL of NaCl (1 M) was added to the solution every 5 min, in ten 20-µL aliquots. After aging overnight at 4 °C, the AuNPs were centrifuged at 6000 rpm for 15 min, and the target AuNPs–DNA was obtained as a clear solution, which was stored in a refrigerator set at 4 °C prior to its use.

### 2.6. SIB Functionalization

SIB functionalization was conducted according to a preparation reported by Wang et al. [34]. First, 2 µL of DNA-c was annealed and allowed to cool to room temperature, then 1 µL of magnetic beads were added and reacted at 37 °C for 1 h. The beads were washed twice with 1 mL of TTL buffer before addition. After the reaction, the beads were washed twice with PBS (1×, pH = 7.4) and suspended in PBS (200 µL) to obtain SIBs–DNA, which was stored at 4 °C. All washing steps were performed under an external magnetic field.

### 2.7. Detection of Target miRNA-21

Next, 0.2-mL microtubes were filled with reaction solutions of different concentrations. Diluted miRNA-21 (1 µL), Exo III (2.5 µL, 2 U/µL), and DNA-a (10 µL, 1 µM) were heated at 37 °C for 1 h and at 95 °C for 10 min to inactivate Exo III, to which an AuNPs–DNA solution (160 µL) was added, mixed evenly, and the mixture was divided into two portions, which were reacted at 37 °C for a further 90 min. After completion of the reaction, one portion was used for fluorescence testing (denoted as *F*), and to the other portion, a solution of functionalized magnetic beads (20 µL) and DNA-d (10 µL, 1 µM) were added, and the reaction was continued for 60 min. Fluorescence testing of the product of this reaction mixture was conducted (denoted as *F*_0_). Finally, a comparison of the fluorescence of each of the reaction portions was performed to measure the difference between the two (*F*_0_ − *F*).

### 2.8. Testing of miRNA-21

A fluorescence spectrometer (RF-6000) was used to measure the fluorescence signal of Texas Red employing a four-pass light quartz cuvette (10 mm) using the experimental solution in PBS (3 mL, 1×). The excitation and emission wavelengths were 580 nm and 620 nm, respectively; the test range was 600–800 nm, the excitation light bandwidth was 5 nm, and the emission light bandwidth was 10 nm. 

## 3. Results and Discussion

### 3.1. Principle of miRNA Detection

**Figure 1:**
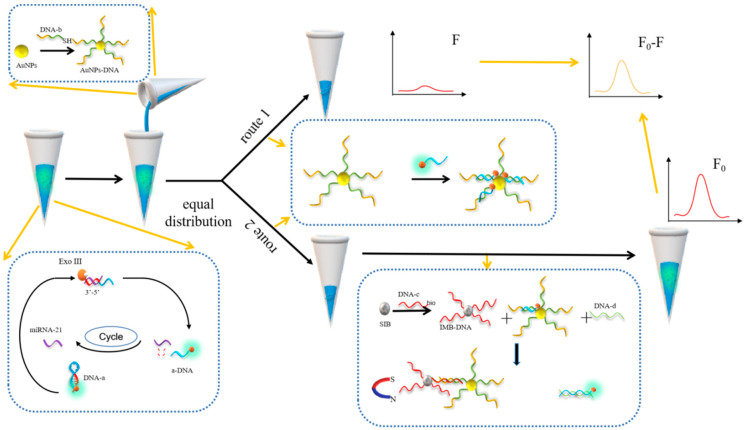
Schematic illustration of the detection of target miRNA-21 using an Exo III-assisted cyclic amplification and AuNPs–DNA quenching fluorophore strategy.

Figure 1 shows the analysis of the mechanism of this sensing system. In this study, hairpin DNA-a was designed and labeled at its 5′ end using a fluorescent Texas Red tag, featuring a miRNA-21 recognition sequence that is partially complementary to DNA-b, DNA-d, and a 3′ end overhang to resist the hydrolysis of Exo III. In the presence of a target detection object, miRNA-21 reacts with DNA-a, and under the action of Exo III, the DNA-a part is hydrolyzed, and a fragment of DNA is released, referred to as a-DNA. Unhydrolyzed miRNA-21 participates in the following reaction to realize a cyclic amplification reaction. AuNP-conjugated DNA-b was added after Exo III inactivation, and the mixture was divided into two equal portions. It can be seen from cycle one that a-DNA reacts with AuNP-conjugated DNA-b to achieve partial hybridization, realize fluorescence quenching, and obtain fluorescence value *F*. In cycle 2, because a-DNA is entirely complementary to DNA-d, and DNA-b is also partially complementary to DNA-c, DNA-d and a-DNA react and hybridize in the presence of DNA-d and DNA-c, DNA-c reacts with DNA-b, and DNA-c functionalized with SIBs attaches to the tube wall under the influence of an external magnet. In this way, separation fluorescence recovery is realized, and fluorescence value *F*_0_ is obtained. Finally, by measuring the difference between the two fluorescence values, it can be determined how much the fluorescence value is shielded (*F*_0_ − *F*) to determine the presence of the miRNA-21 analyte.

### 3.2. Characterization of the AuNPs

**Figure 2:**
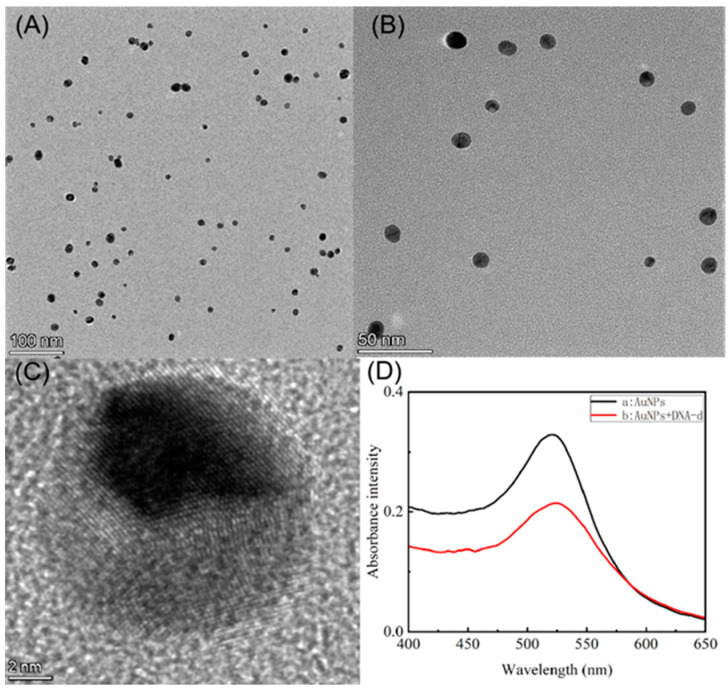
(**A**–**C**) TEM images of the AuNPs ((**A**–**C**) show the TEM images at 100 nm, 50 nm, and 2 nm, respectively); (**D**) UV spectra of AuNPs (black curve a) and AuNPs–DNA (red curve b), with curve b red-shifted by 3 nm with respect to curve a.

The structure and size of the AuNPs were measured using TEM, and the absorbance of the AuNPs was measured by UV spectroscopy, with the results shown in Figure 2. From the TEM images shown in Figure 2A–C, it can be concluded that AuNPs were successfully synthesized with a uniform dispersion, with the majority of the particles measuring 16 ± 4 nm in size. The absorbance of the AuNPs is observed at 524 nm by UV spectroscopy, whereas the AuNPs–DNA exhibit a clear 3-nm red-shifted absorption peak at 521 nm (Figure 2D), which is related to the change in the local surface plasmon resonance effect, indicating the successful DNA modification of the AuNPs. At the same time, in order to verify whether the synthesized AuNPs-DNA can quench the fluorescence through FRET, we compared the excitation spectra of the same concentration of fluorescent marker Texas Red before and after the reaction, as shown in Figure S1, the two curves are surrounded by the area of AuNPs is the overlapping part of AuNPs absorbing Texas Red fluorescence, and the overlapping area is 78.32% after calculation.

### 3.3. Feasibility Study of the miRNA Assay

**Figure 3:**
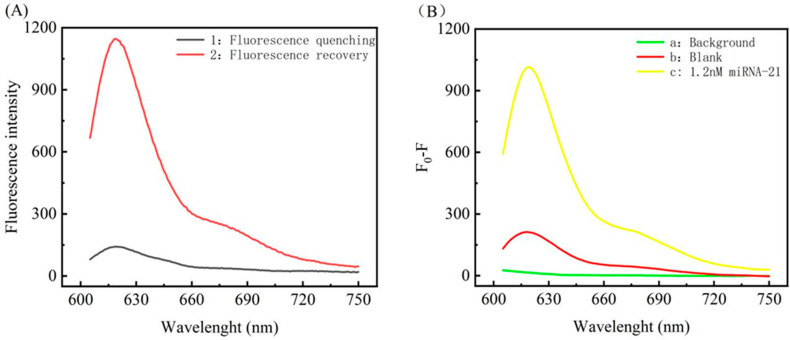
(**A**) Fluorescence emission spectra of the system under different conditions. Curve 1 shows the burst effect of AuNPs–DNA on fluorescence in the reaction system in the presence of miRNA-21. Curve 2 shows the fluorescence recovery upon adding DNA-d and IMB under the same conditions as those of curve 1. (**B**) Effect of the system on fluorescence under different conditions. a shows the background signal of the reaction system, b shows the effect on fluorescence in the absence of miRNA-21, and c shows the effect of AuNPs–DNA on fluorescence in the presence of miRNA-21.

The feasibility of this strategy was verified by detecting the fluorescence response signals of the system before and after adding the detection target. After adding the target miRNA-21, through the reaction hybridization with DNA-a in the reaction system, the fragmented DNA of DNA-a was obtained under the hydrolysis of Exo III, referred to as a-DNA. a-DNA again reacts with AuNPs–conjugated DNA-b, resulting in the quenching of the experimental fluorescence, shown as curve 1 in Figure 3A. The results show that the fluorescence (*F*) of the system is significantly reduced, indicating successful fluorescence labeling. AuNP-conjugated DNA-b successfully quenches the compound; under the reaction conditions of curve 1, DNA-d, which is entirely complementary to a-DNA, and DNA-c, which reacts with AuNPs–DNA and conjugates to SIBs, respectively, were added. The results were as follows. Figure 3A curve 2 indicates that fluorescence recovery (*F*_0_) was achieved. By comparing the difference (*F*_0_ − *F*) between curves 1 and 2 shown in Figure 3A, curve a in Figure 3B was obtained, from which it can be seen that the fluorescence is significantly increased in the presence of the target miRNA-21. Under the same conditions, curve b in Figure 3B was obtained in the absence of miRNA-21. It can be seen that there is little change in the fluorescence, as Exo III does not hydrolyze DNA-a in the absence of miRNA-21, so when DNA-a forms a hairpin structure by itself, it becomes difficult for it to react with AuNPs–DNA, thus there is little change in fluorescence. The little difference in the signals is mainly due to the influence that the AuNPs in the system have on the unreacted DNA-a and the experimental error experienced during operation; see Figure S2 for details. In conclusion, the qualitative detection of target miRNA-21 can be achieved by comparing the difference in fluorescence of the two reactions, indicating that the method is feasible.

### 3.4. Optimization of the Experimental Conditions

Hybridization of NP–DNA probes to target–DNA is a sensitive step in the design of nanobiosensors. Therefore, an effective parameter for evaluating fluorescence intensity is necessary. In this study, factors such as AuNPs–DNA dosage, temperature, Exo III dosage, and hybridization time were studied. When one variable was changed, the other variables remained unchanged. A DNA-a (1 µM) dosage of 10 µL, a SIBs–DNA dosage of 20 µL, and a DNA-d (1 µM) dosage of 10 µL were used, with the results of the study as follows.

#### 3.4.1. AuNPs–DNA Optimization

**Figure 4:**
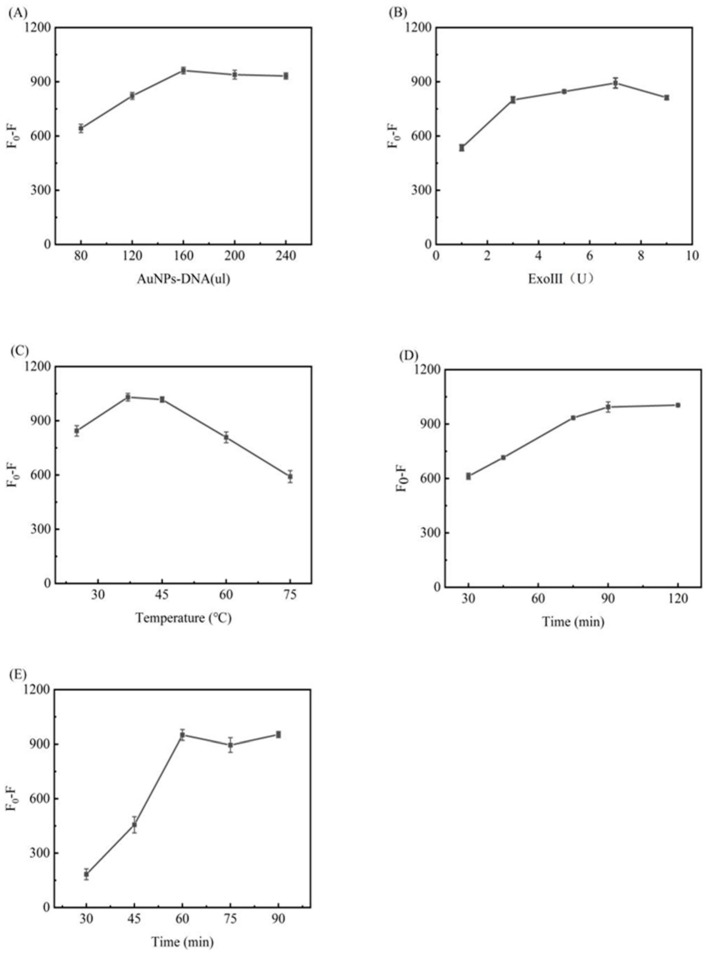
Optimization of the experimental conditions: (**A**) volume of AuNPs–DNA added; (**B**) concentration of Exo III added; (**C**) change in the reaction temperature; (**D**,**E**) optimization of the reaction time, corresponding to fluorescence quenching, where (**D**) shows the extinction stage and (**E**) shows the fluorescence recovery stage.

The amount of AuNPs–DNA is related to the shielding effect on the fluorescence. The conditions were optimized by determining the fluorescence value, which will neither lead to a decrease in the signal-to-noise ratio nor too little, resulting in an unsatisfactory shielding of the fluorescence value. Figure 4A shows the experimental results, from which it can be seen that with an increase in the amount of AuNPs–DNA, the difference in fluorescence (*F*_0_ − *F*) gradually increases and reaches a maximum value at 160 µL, with very little change in the fluorescence upon an increase in the amount of AuNPs–DNA. Therefore, the dosage of AuNPs–DNA used was 160 µL.

#### 3.4.2. Exo III Optimization

After determining the amount of DNA-a and miRNA-21, the amount of Exo III is related to whether the DNA-a can be fully hydrolyzed and thus affects the subsequent experiments, such as reaction efficiency and signal output. After analysis of the experimental results, as shown in Figure 4B, the difference in fluorescence (*F*_0_ − *F*) was found to increase with an increase in the Exo III dosage, reaching a maximum at 5 U. A further increase affected the miRNA-21 concentration under the same conditions. Therefore, the amount of Exo III used was 5U.

#### 3.4.3. Temperature Optimization

Temperature is one of the critical factors that affect fluorescence intensity. Too low a temperature increases the reaction time, and too high a temperature affects the activity of Exo III, thus affecting the experiment. The experimental results are shown in Figure 4C, from which it can be seen that the difference in fluorescence (*F*_0_ − *F*) increases in the range of 25–37 °C, shows little change in the range of 37–45 °C, and decreases in the range of 45–75 °C. Therefore, 37 °C was chosen as the optimal reaction temperature.

#### 3.4.4. Time Optimization

The optimization of time is divided into two parts. The first part is related to a-DNA and the AuNPs–DNA reaction time, which is the process of fluorescence burst; this process only changes *F* after the burst and does not change *F_0_*. After the experiments, the results shown in Figure 4D show that in the range of 30–90 min, *F*_0_ − *F* shows an increasing trend, reaching a maximum of 90 min, with no changes observed after 90 min. Therefore, 90 min was chosen as the optimal time. The second part of the process does not change the fluorescence value after burst (*F*), but changes *F*_0_, that is, the fluorescence recovery phase, as shown in Figure 4E, with the optimal reaction time being 60 min.

### 3.5. Assay Performance for miRNA-21 Detection

**Figure 5:**
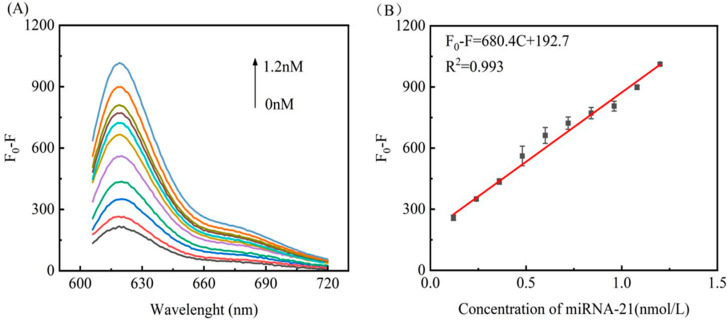
(**A**) Fluorescence emission spectra of miRNA-21 at different concentrations. The curves from the bottom to the top represent 0.0 nM, 0.12 nM, 0.24 nM, 0.36 nM, 0.48 nM, 0.60 nM, 0.72 nM, 0.84 nM, 0.96 nM, 1.08 nM, and 1.20 nM targets. (**B**) Linear relationship of *F*_0_ – *F* and the concentration of miRNA-21 in the range of 0.12–1.2 nM. The error bars represent the standard deviation of triplicate measurements.

The purpose of this study was to design a biosensor based on Exo III hydrolysis, magnetic bead separation, and the fluorescence shielding of AuNPs for the sensitive and rapid detection of target miRNA-21. The optimized experimental conditions were an AuNPs–DNA dosage of 160 μL, Exo III dosage of 5 U, reaction temperature of 37 °C, and reaction time of 90 min in the fluorescence quenching stage and 60 min in the fluorescence recovery stage. The maximum difference in fluorescence signal (*F*_0_ − *F*) is observed using these optimized conditions. Using the optimized conditions and the same experimental steps, the designed detection system was used to detect target difference concentrations of miRNA-21 to explore the sensitivity of the sensor to target miRNA and to establish a relationship between miRNA-21 and the fluorescence intensity at different concentrations. Each experiment was repeated three times, with the experimental results shown in Figure 5A. In the range of 0−1.2 nmol L^−1^, with a gradual increase in the miRNA-21 concentration, the difference in the fluorescence intensity (*F*_0_ − *F*) gradually increased. At a target detector concentration of as low as 0.12 nM, the fluorescence intensity of this concentration was still quite different from that of the non-target concentration, indicating that the detection strategy exhibits high sensitivity. Figure 5B shows the relationship between the relative fluorescence intensity (*F*_0_ − *F*) at 580 nm and the concentration from 0.12–1.2 nmol L^−1^. After simple fitting, a good linear relationship is observed in the range of 0.12–1.2 nmol L^−1^ of *F*_0_ − *F*= 680.4C + 192.7 (R^2^ = 0.993). The limit of detection (LOD) was based on three times the standard deviation (3σ) of the blank signal divided by the slope, calculated to be 18 pM. The experimental results show that this method has a good linear range and detection limit.

### 3.6. Repeatability and Longtime Stability of Biosensor

**Figure 6:**
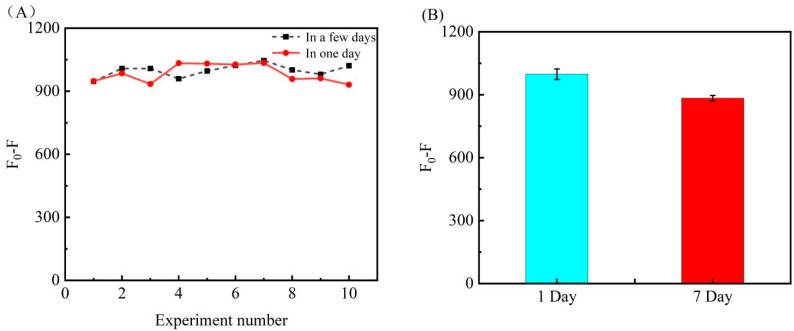
(**A**) is a dot-line graph obtained from repeated experimental data, the black in the figure is the results of 10 groups of experiments carried out on different days; the red curve is the results of 10 groups of experiments carried out in one day. (**B**) In order to test the stability of the bioconjugate (AuNPs-DNA and SIBs-DNA), the measured data were tested three times. The left side is the experimental result of the first day, and the right side is the seventh day of the experiment. The results obtained.

In order to verify that the experimental results tested by the experimental method of this experiment are a repeatable process, we conducted 10 sets of experimental tests on a single day, and a total of 10 sets of experiments on different days. The experimental results are shown in Figure 6A, and the results show that the results obtained from these 20 sets of experiments are all within a general range, which indicates that the sensor constructed for this experiment is experimentally reproducible. To further explore the reproducibility, we processed the experimental data by substituting the linear equation *F*_0_ − *F* = 680.4C + 192.7 (R^2^ = 0.993) with 1.2 nM as the test concentration and performed a standard t-test with a confidence level of 95% (alpha = 0.05) (see Table S2 in the Supplementary Materials for specific data analysis), and obtained the results p1 = 0.305 > 0.05 and p2 = 0.100 > 0.05 with confidence intervals of [980.07, 1017.52] and [957.45, 1010.96] respectively were not significantly different, indicating excellent reproducibility of the experimental results. We also conducted experiments on the storage stability of the bioconjugates (AuNPs-DNA and SIBs-DNA), and the results were obtained by placing the prepared biocouples for 7 days after the experiments and comparing them with the results of the first day, as shown in Figure 6B. The results showed that the experimental results after 7 days were 88.6% of the experimental results on the first day, indicating that the AuNPs-DNA and SIB functionalized substances had longer storage stability.

### 3.7. Specificity Analysis

**Figure 7:**
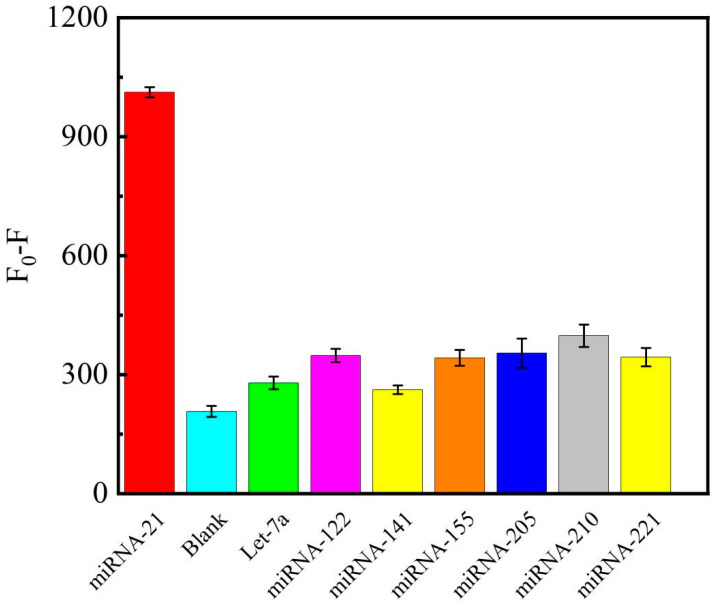
Relative fluorescence intensity at an emission wavelength of 580 nm versus different miRNA species, where the error bars represent the standard deviation of three replicate experiments.

Further experiments were conducted to test the detection accuracy of this experimental sensor toward miRNA-21. Under the same optimized conditions, the difference in the fluorescence (*F*_0_ − *F*) measured by detecting other homologous miRNAs similar to miRNA-21 was investigated. Theoretically, if there are mismatched or non-target miRNAs, the efficiency of reacting with DNA-a and driving the following reaction will be reduced, eventually leading to changes in fluorescence intensity. Under the same optimized conditions, to demonstrate the integrity of the experiments, multiple miRNA selections were selected for specific detection (miRNA-141, miRNA-210, miRNA-221, let-7a, miRNA-205, and miRNA-16). Under five times the concentration of other substances, the experimental results shown in Figure 7 were obtained. Detailed fluorescence curves are shown in Figure S3. It can be seen from the histogram that under the same conditions, the detection results of miRNA-21 show excellent selectivity compared with other miRNAs and the control group. Its difference in fluorescence (*F*_0_ − *F*) is much higher than those of the other miRNAs, indicating that this detection strategy exhibits reasonable specificity and selectivity.

### 3.8. Determination of Target DNA in Human Serum Samples

To test the stability and sustainability of the sensing system in a complex environment, the applicability of the developed method was validated in actual human serum samples. By adding different concentrations of targets to complex bioenvironmental systems with serum, the recovery of three different concentrations of the target DNA (0.25 nM, 0.4 nM, and 0.5 nM) was performed in 10% human serum samples in PBS buffer. The recoveries were calculated to be in the range of 90.8–100.4%, as shown in Table 1. And the t-test was used for the obtained data to find that the *p*-values were all greater than 0.05, indicating that the results were within the range of the 95% confidence level (the specific numerical analysis is in the Supplementary Materials Table S3). The relative standard deviation recoveries of 6.4%, 1.4%, and 0.4%, for 0.25 nM, 0.4 nM, and 0.5 nM of the target miRNA-21, respectively, with these recoveries meeting the requirements of the experiment. The smaller the standard deviation, the more similar the test results are to the expected results, and the better the precision and accuracy of the method. The experimental results thus indicate that the proposed assay in this study has the potential to detect miRNA-21 in actual complex biological samples.

## 4. Conclusions

A sensing analysis strategy was established for the sensitive detection of miRNA-21 at as low as 18 pM via Exo III-assisted cyclic amplification, excellent separation by SIBs, and fluorescence burst by AuNPs–DNA. In the detection of the target miRNA-21, signal amplification was achieved by Exo III in the presence of miRNA-21, and an excellent fluorescence burst was achieved by the AuNPs, with the signal recovery achieved by the effect of the separation of the SIBs. This strategy has the following advantages. First, it greatly reduces the detection limit by indirectly detecting enzymatic cycle amplification. Second, the fluorescence difference *F*_0_ − *F* is obtained by two tests in one set of experiments, which can be compared from *F*, *F_0_*, and *F*_0_ − *F* in the case of multiple experiments in practical applications, improving the accuracy of the assay. Thirdly, this strategy provides a simple, rapid, and low-cost platform for miRNA-21 detection, showing its good application prospects. In addition, this strategy can be applied in detecting other disease-related biomarkers with reasonable specificity, stability, and utility by modifying the target recognition sequence in DNA-a, providing a convincing reference for the early diagnosis of related cancers.

## Figures and Tables

**Table 1:** Recovery of miRNA-21 in a spiked human serum sample (*n* = 3).

Sample	Added (nM)	Found (nM)	Recovery (%)	RSD (%)
1	0.24	0.218 0.221 0.244	90.8 95.4 95.5	6.4
2	0.54	0.538 0.525 0.525	97.9 95.5 95.5	1.4
3	0.75	0.747 0.753 0.750	99.6 100.4 100.0	0.4

## Data Availability

Not applicable.

## Supplementary Materials

The following supporting information can be downloaded at: https://www.mdpi.com/article/10.3390/bios12090724/s1, Figure S1: Excitation spectra at 620 nm before and after the reaction of AuNPs-DNA and Texas Red; Figure S2: Effects of AuNPs-DNA and SIBs-DNA on fluorescence in the absence of miRNA-21; Figure S3: Experimental results Fluorescence values corresponding to various miRNAs; Table S1: Synthesized Oligonucleotides sequences employed in this work; Table S2: *t*-test analysis table; Table S3: The *t*-test in the practical application of human serum.
