# Supplementary material for: Gold Nanoparticle-Based Enzyme-Assisted Cyclic Amplification for the Highly-Sensitive Detection of miRNA-21
Source: Biosensors (Basel). 2022 Sep 4;12(9):724. doi: 10.3390/bios12090724 (PMC9496089; doi:10.3390/bios12090724)
Supplement: Supplementary file 1 [file biosensors-12-00724-s001.zip › biosensors-1883334-supplementary.pdf]

Supplementary Materials

Gold Nanoparticle-based Enzyme-assisted Cyclic Amplification for the Highly-sensitive Detection of miRNA-21

Yang Qing, Yuxing Yang, Ping Ouyang, Chenxin Fang, Haobin Fang, Yazhen Liao, Haiyu Li ,Zhencui Wang, Jie Du*

State Key Laboratory of Marine Resource Utilization in South China Sea, College of Materials Science and Engineering, Hainan University, Haikou 570228, China

*Corresponding author: dujie@hainanu.edu.cn

1. Materials

Table S1 Synthesized Oligonucleotides sequences employed in this work

Oligonucleotide	Sequence
DNA-a	5'-Texas Red-ATGTTGATACGATTCCGGCGCAATCAACATCAGTCTGATAAGCTA-3'
DNA-b	5'-CGGGCCGAATCGTATAACCATTTT- C6 SH-3'
DNA-c	5'-CCCGGCCCGTTTTTTTTTTTTTTT -Biotin-3'
DNA-d	5'-TTGCGCCGGAATCGTATCAACAT-3'
MiRNA-21	UAG CUU AUC AGA CUG AUG UUG A
MiRNA-16	UAG CAG CAC GUA AAU AUU GGC G
MiRNA-141	UAA CAC UGU CUG GUA AAG AUG G
MiRNA-205	UCC UUC AUU CCA CCG GAG UCU GU
Let-7a	UGA GGU AGU AGG UUG UAU AGU U
MiRNA-221	AGC UAC AUU GUC UGC UGG GUU UC
MiRNA-210	CUG UGC GUG UGA CAG CGG CUG A

2 Characterization of AuNPs

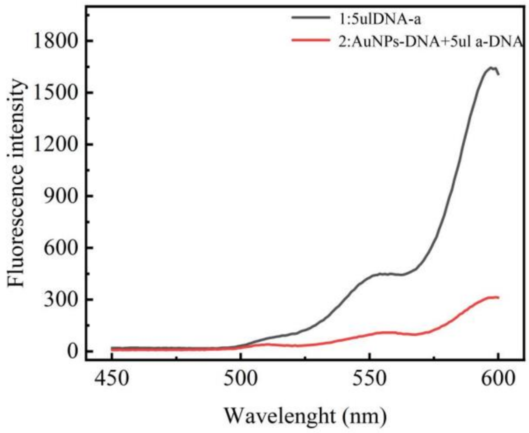

Figure S1. Excitation spectra at 620 nm before and after the reaction of AuNPs-DNA and Texas Red.

3. Feasibility study of the miRNA assay

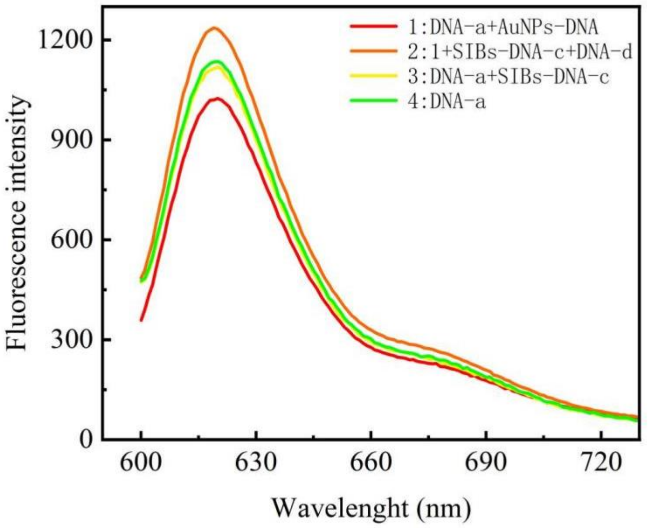

Figure S2. Effects of AuNPs-DNA and SIBs-DNA on fluorescence in the absence of miRNA-21.

4. Repeatability and longtime stability of biosensor

Table 2 is a t-test analysis table, which is analyzed by standard t-test results with a confidence level of 95% (alpha=0.05) on the data measured within one day and within a few days. The 10 groups of fluorescence data tested within one day are 946, 1008, 1008, 959, 996, 1023, 1046, 1001, 980, 1021; the 10 groups of data tested within a few days are 948, 985, 934, 1033, 1031, 1027 , 1034, 958, 961, 931.The selected linear regression equation was substituted into the linear equation F_0-F=680.4C+192.7 (R^2=0.993) with 1.2 nM as the test concentration, and the theoretical average value was 1009.18, the target number was 10, and the t value, P value and confidence interval were calculated. The results are shown in Table 2, and the P values are all greater than 0.05. The results show that the experiment is repeatable, and the two confidence intervals are not much different, indicating that the experimental results are within a certain range and have certain stability.

Table S2 T-test analysis table. On the left is the analysis of 10 groups of experimental data in one day. On the right are 10 sets of experimental data analysis within a few days.

In one day（Flourescence intensity）t-test	In a few days（Flourescence intensity）t-test
α=0.05	α=0.05
sample average=998.8	sample average=984.2
null average=1009.18	null average=1009.18
standard delviation=30.2096379	standard delviation=43.16583011
N=10	N=10

t_1=1.086555298	t_2=1.830005255
P_1=0.305476152	P_2=0.100494771
confidence interval=[980.07, 1017.52]	confidence interval=[957.45, 1010.96]

5. Specificity analysis

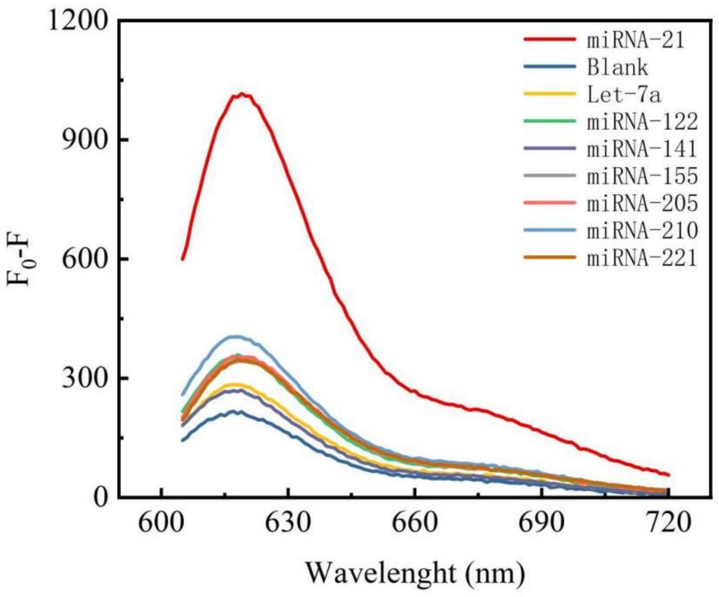

Figure S3. Experimental results Fluorescence values corresponding to various miRNAs.

6. Determination of target DNA in human serum samples

Table S3 The t-test in the practical application of human serum.The p-values of the experimental results are all greater than 0.05, indicating that the 95% confidence interval is within the 95% confidence interval, indicating that it also has good feasibility in the practical application of human serum.

Sample	Added (nM)	Found (nM)	t	P
1	0.24	0.218<br>0.221<br>0.244	1.502	0.272
2	0.54	0.538<br>0.525<br>0.525	2.462	0.133
3	0.75	0.747<br>0.753<br>0.750	0	1
